# Efficacy and safety of risedronate 150-mg once a month in the treatment of postmenopausal osteoporosis: 2-year data

**DOI:** 10.1007/s00198-012-2056-0

**Published:** 2012-06-30

**Authors:** M. R. McClung, J. R. Zanchetta, A. Racewicz, C. Roux, C.-L. Benhamou, Z. Man, R. A. Eusebio, J. F. Beary, D. E. Burgio, E. Matzkin, S. Boonen, P. Delmas

**Affiliations:** 1 Oregon Osteoporosis Center, 5050 NE Hoyt, Suite 626, Portland, OR 97213 USA; 2 Instituto de Investigaciones (IDIM), Buenos Aires, Argentina; 3 Centrum Medyczne Specjalistyczny Gabinet Lekarski, Bialystok, Poland; 4 Paris Descartes University, Cochin Hospital, Paris, France; 5 INSERM Research Unit U658, Orléans, France; 6 Centro TIEMPO, Buenos Aires, Argentina; 7 Procter and Gamble Company, Mason, OH USA; 8 The sanofi-aventis Group, Bridgewater, NJ USA; 9 Bone Research Unit, Department of Experimental Medicine, Katholieke Universiteit Leuven, Leuven, Belgium; 10 INSERM Research Unit 831, University of Lyon, Lyon, France

**Keywords:** Bone mineral density, Fracture risk, Monthly, Osteoporosis, Risedronate

## Abstract

**Summary:**

This study showed that risedronate 150-mg once a month provides similar efficacy and safety at 2 years compared with risedronate 5-mg daily for the treatment of postmenopausal osteoporosis. This adds to the range of risedronate dosing options and provides an alternative for patients who prefer once-a-month dosing.

**Introduction:**

Risedronate is effective in the treatment of postmenopausal osteoporosis in oral daily, weekly, or on two consecutive days per month doses. This 2-year randomized, double-blind, multicenter study assesses the efficacy and safety of a single risedronate 150-mg once-a-month oral dose compared with the 5-mg daily regimen.

**Methods:**

Women with postmenopausal osteoporosis were randomly assigned to receive risedronate 5-mg daily (*n* = 642) or 150-mg once a month (*n* = 650) for 2 years. Bone mineral density (BMD), bone turnover markers, new vertebral fractures, and adverse events were evaluated. The primary efficacy endpoint was the mean percent change from baseline in lumbar spine BMD after 1 year.

**Results:**

Four hundred ninety-eight subjects in the daily group (77.6 %) and 513 subjects in the once-a-month group (78.9 %) completed the study. After 24 months, the mean percent change in lumbar spine BMD was 3.9 % (95 % confidence interval [CI], 3.43 to 4.42 %) and 4.2 % (95 % CI, 3.68 to 4.65 %) in the daily and once-a-month groups, respectively. The once-a-month regimen was determined to be non-inferior to the daily regimen. The mean percent changes in BMD at the hip were similar in both dose groups, as were changes in biochemical markers of bone turnover. The incidence of adverse events, adverse events leading to withdrawal, and upper gastrointestinal tract adverse events were similar in the two treatment groups.

**Conclusions:**

After 2 years, treatment with risedronate 150-mg once a month provided similar efficacy and tolerability to daily dosing and provides an alternative for patients who prefer once-a-month oral dosing.

## Introduction

Risedronate is a pyridinyl bisphosphonate that has been shown in prospective studies to reduce the risk of vertebral, nonvertebral, and hip fractures [1–3]. Like other bisphosphonates, risedronate remains active on the surface of bone for long periods after dosing, providing the opportunity to develop a range of dosing schedules.

The original risedronate dosing regimen for postmenopausal osteoporosis was an oral dose of 5-mg daily [1–3]. It was later demonstrated that risedronate 35-mg once a week and 75-mg each day for two consecutive days a month provided similar efficacy and safety to the daily regimen [4, 5]. The efficacy and tolerability of risedronate once-a-month dosing (150-mg) was compared with risedronate daily dosing (5-mg) in women with osteoporosis with changes in lumbar spine bone mineral density (BMD) as the primary endpoint. After 1 year of treatment, published previously, the efficacy of risedronate 150-mg once-a-month regimen was non-inferior to the 5-mg daily regimen [6]. The once-a-month regimen also had a similar tolerability profile as the daily regimen after 1 year of treatment. This study continued for an additional year of treatment, and the results of the complete study over 2 years are presented here.

## Materials and methods

### Study design

This randomized, double-blind, active-controlled, parallel-group non-inferiority study was conducted at 47 study centers in the Americas, Europe, Australia, and Asia (Appendix). The first subject was screened in October 2005, and the last subject observation took place in March 2008. The study was performed in accordance with good clinical practice and the ethical principles that have their origin in the Declaration of Helsinki. The protocol was approved by the appropriate institutional review boards or ethics committees, and the subjects gave written, informed consent to participate.

### Patients

Eligible subjects who gave consent were randomly assigned in a 1:1 ratio to the two treatment groups. Women were eligible to enroll in the study if they were at least 50 years of age, ambulatory, in generally good health, postmenopausal (at least 5 years since last menses), had at least three vertebral bodies in the lumbar spine (L1 to L4) that were evaluable by densitometry (i.e., without fracture or degenerative disease), and had a lumbar spine BMD T-score of less than −2.5 or a T-score of less than −2.0 with at least one prevalent vertebral fracture (T4 to L4). Specific details of the inclusion criteria and methods have been previously published [6].

### Treatments

Subjects received oral risedronate 5-mg daily or 150-mg once a month (i.e., a single 150-mg tablet on the same calendar day each month, followed by a placebo tablet daily for the rest of the month). All tablets were identical in appearance and supplied in identical blister cards. Tablets were taken on an empty stomach in the morning at least 30 min before the first food or drink of the day, with at least 4 oz of plain water. Subjects were instructed to remain in an upright position for at least 30 min after dosing. Subjects were considered compliant if they took at least 80 % of the study tablets. Calcium (1,000-mg/day) and vitamin D (400–500 IU/day) were supplied to all subjects, although they were allowed to take up to 1,000 IU/day of vitamin D. These supplements were taken with a meal other than breakfast and not with the study medication.

### Efficacy assessments

Dual x-ray absorptiometry (DXA) measurements of the lumbar spine and proximal femur were obtained at baseline and after 6, 12, and 24 months using instruments manufactured by Lunar Corporation (General Electric, Madison, WI, USA) or Hologic (Waltham, MA, USA). DXA scans collected at the clinical sites were sent to a central facility for quality control and analysis (Synarc, Copenhagen/Hamburg). Lateral thoracic and lumbar spine radiographs collected at screening and at 12 and 24 months were analyzed for vertebral fractures by semi-quantitative analysis [7] at a central radiology site (Synarc, Copenhagen/Hamburg). Biochemical markers of bone turnover were assessed at 3, 6, 12, and 24 months. Serum bone-specific alkaline phosphatase (BALP) was measured using an immunochemiluminescence assay on an automatic analyzer (Ostase, Access, Beckman Coulter, LaBrea, CA, USA). The intra- and interassay coefficients of variation for this measurement were less than 4 and 10 %, respectively. The detection limit of the test was 0.07 ng/mL, and the limit of quantitation was 0.28 ng/mL. Urinary N-terminal crosslinking telopeptide of type I collagen (NTX) was measured with an electrochemiluminescent immunoassay on an automated machine (Vitros ECi, Johnson and Johnson, Rochester, NY, USA). The intra- and interassay coefficients of variation were below 7 and 6 %, respectively. The detection limit of the test was 4 nM, and the limit of quantitation was 22 nM. This measurement was corrected for creatinine (NTX/Cr). Serum C-terminal crosslinking telopeptide of type I collagen (CTX) was measured using an enzyme immunoassay kit (Serum CrossLaps^®^, Nordic Bioscience Diagnostics, Herlev, Denmark). The intra- and interassay coefficients of variation were below 8 and 6 %, respectively. The lower limit of detection was 0.044 ng/mL. Bone turnover marker assays were performed at a central laboratory (Synarc SAS, Lyon, France). The samples for the 24-month study visit were measured at a different time than the samples for all previous visits.

### Safety assessments

Physical examinations were performed at baseline and after 12 and 24 months. Vital signs, concomitant medications, and adverse event reports were recorded at regular clinic visits throughout the study. Adverse event reports were captured using the Medical Dictionary for Regulatory Activities (MedDRA) system. Blood and urine samples for clinical chemistry and other standard laboratory measurements were collected at baseline and after 3, 6, 9, 12, 18, and 24 months of treatment. Specimens were analyzed by Quintiles Laboratories (Smyrna, GA, USA).

### Statistical analysis

The primary endpoint analysis was a test of non-inferiority comparing the least squares mean percent change from baseline in lumbar spine BMD in the 150-mg once-a-month and 5-mg daily groups after 12 months. This test employed a predefined non-inferiority margin of 1.5 % and a one-sided type I error of 2.5 %. The results of this analysis have been published previously [6].

Secondary endpoints included the percent change from baseline in lumbar spine BMD at months 6 and 24, and at endpoint; the percent change from baseline in BMD of the total proximal femur, femoral neck, and femoral trochanter at months 6, 12, and 24, and at endpoint; the percentage of patients with new vertebral fractures at year 1 and 2; and the percent change from baseline in biochemical markers of bone turnover (NTX/Cr, CTX, and BALP) at months 3, 6, 12, and 24, and at endpoint. All data reported here are based upon cumulative data collected over the entire 2-year treatment period.

After 2 years of treatment, a non-inferiority analysis was performed based on the one-sided 97.5 % confidence interval (CI) for the difference in mean percent change from baseline to month 24 in lumbar spine BMD. The CIs were constructed using an ANOVA model with fixed effects for treatment and pooled investigative center. If the upper bound of the 97.5 % one-sided CI did not exceed 2.0 %, then the once-a-month treatment was considered non-inferior to the daily treatment. This analysis was based on all subjects who were randomized and took at least one dose of study medication and who had evaluable lumbar spine BMD measurements at both baseline and at least one postbaseline time point (last observation carried forward; this is the intent-to-treat [ITT] population). The month 24 non-inferiority “delta” was selected using the same rationale used to select the month 12 non-inferiority margin. In previous studies contrasting risedronate 5-mg daily and placebo for the treatment of postmenopausal osteoporosis, the mean percent change difference between the treatment groups in lumbar spine BMD from baseline to month 24 ranged from 4.1 to 5.4 %. Thus, using a “delta” of 2.0 % would maintain approximately 50 % of the effect size of the risedronate 5-mg daily dose relative to placebo at month 24.

The treatment group differences at month 24 in percent changes in proximal femur BMD and bone turnover markers were analyzed using an ANOVA model; two-sided 95 % CIs for the treatment differences were constructed using the ITT population. The incidence of new vertebral fractures over 24 months was analyzed using Fisher's exact test. Adverse events were summarized as frequency distribution tables and reported by treatment group.

## Results

### Subjects

**Fig. 1:**
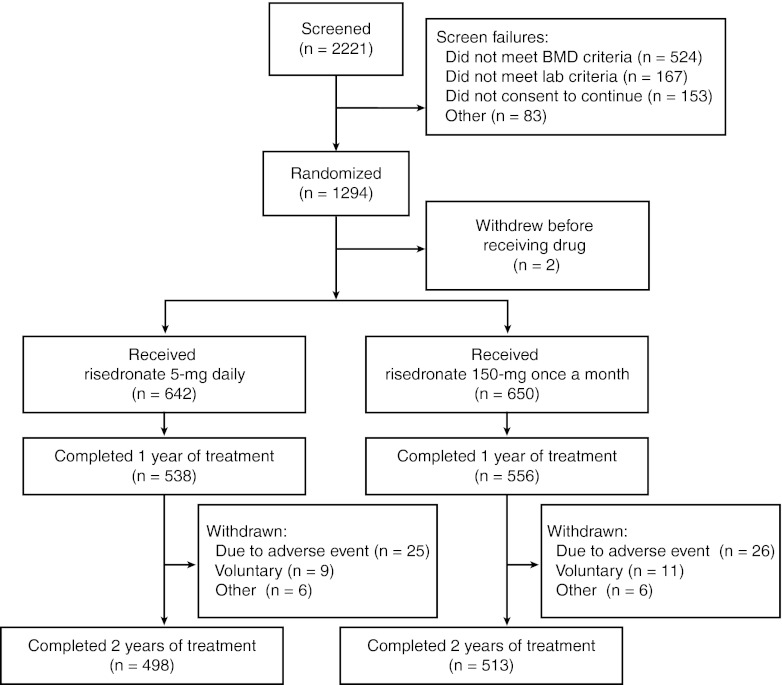
Disposition of subjects. *BMD* bone mineral density

From the total of 2,221 women who were screened, 1,294 subjects were randomized, and 1,292 subjects received at least one dose of study drug (Fig. 1). Overall, baseline characteristics were similar in both treatment groups. Demographics of the subjects in each treatment group have been reported previously [6]. A similar percentage of subjects in each treatment group completed 24 months of the study (5-mg daily group, 77.6 %; 150-mg once-a-month group, 78.9 %). The most common reasons given for withdrawal during year 2 were adverse event and voluntary withdrawal, which occurred at similar incidences in both treatment groups. A high percentage of subjects in both groups (95.5 % of subjects in the 5-mg daily group and 95.7 % of subjects in the 150-mg once-a-month group) took at least 80 % of the study tablets.

### Efficacy assessments

**Fig. 2:**
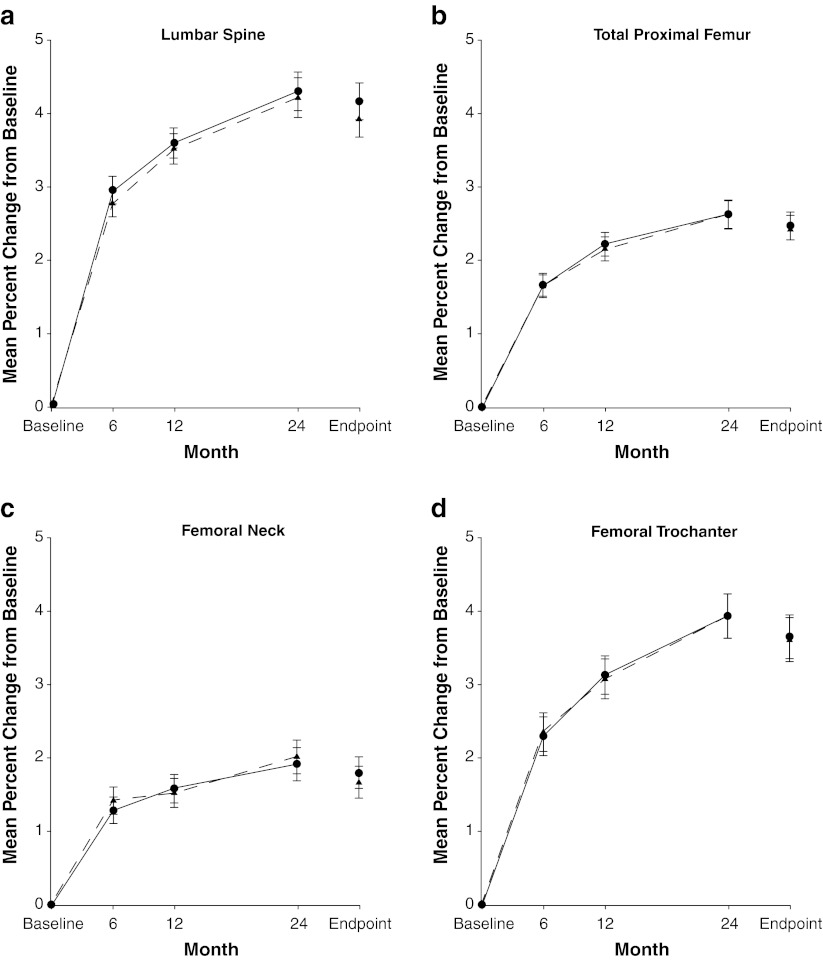
Mean percent change (±SEM) from baseline in bone mineral density in women receiving risedronate 5-mg daily (*dashed line with triangles*) or 150-mg once a month (*solid line with circles*). Endpoint refers to the value calculated using the last observation carried forward at month 24. There were no statistically significant differences between treatment groups at any time point at any of these sites. *OAM* once a month

The within-group mean percent changes from baseline in lumbar spine BMD were statistically significant in both treatment groups at each time point (Fig. 2). The mean percent changes at 24 months (95 % CI) were 3.9 % (3.43 to 4.42 %) for the 5-mg daily group and 4.2 % (3.68 to 4.65 %) for the 150-mg once-a-month group. The difference from the 5-mg daily group (daily minus once a month) in mean percent change from baseline in lumbar spine BMD at month 24 was –0.24 % (95 % upper confidence bound, 0.25 %). This upper boundary was well below the 2.0 % needed to establish non-inferiority; thus, the 150-mg once-a-month regimen was determined to be non-inferior to the 5-mg daily regimen at 24 months. Significant increases from baseline in BMD were observed at 6, 12, and 24 months in both treatment groups (Fig. 2). There was no statistically significant difference between treatment groups in mean percent change in BMD at the lumbar spine or regions of the proximal femur (total proximal femur, femoral neck, and femoral trochanter) at any time point.

There was no difference between treatment groups in the occurrence of new incident vertebral fracture as determined by morphometric measurement during the study; 14 subjects (2.5 %) in the 5-mg daily group and 15 subjects (2.6 %) in the 150-mg once-a-month group experienced such a fracture.

**Fig. 3:**
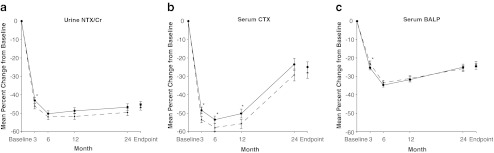
Mean percent change (±SEM) from baseline in biochemical markers of bone turnover in women receiving risedronate 5-mg daily (*dashed line with triangles*) or 150-mg once a month (*solid line with circles*). Endpoint refers to the value calculated using the last observation carried forward at month 24. *CTX* C-terminal crosslinking telopeptide of type I collagen, *NTX* N-terminal crosslinking telopeptide of type I collagen, *OAM* once a month. *p < 0.05 indicates a statistically significant difference between treatment groups (unadjusted for multiple comparisons)

Significant decreases from baseline in NTX/Cr, CTX, and BALP were observed at 3, 6, 12, and 24 months in both treatment groups (Fig. 3). In general, changes from baseline in these biochemical markers were similar in both treatment groups. The small difference in CTX between groups was statistically significant at months 3, 6, and 12 but not at month 24. There was no statistically significant difference between treatment groups at endpoint (the last-observation-carried forward values at month 24) for any of the biochemical markers of bone turnover.

### Safety assessments

Overall, the frequency of adverse events was similar in both treatment groups (Table 1). Among the most common adverse events, only diarrhea and influenza were more frequent in the monthly group compared with the daily group after 24 months. The difference between groups in these adverse events was primarily driven by events reported during the first few months of the study. Most events of diarrhea were mild or moderate in severity. One subject (0.2 %) in the 5-mg daily group and six subjects (0.9 %) in the 150-mg once-a-month group withdrew from the study as a result of diarrhea. All events of influenza were mild or moderate in severity, most occurred more than 90 days after the start of treatment, and none of the subjects withdrew because of influenza. More patients in the 150-mg once-a-month group reported serious adverse events than in the 5-mg daily group (Table 1). This difference was predominantly due to small differences between the two groups in several MedDRA System Organ Class categories, and none of the differences in any of the individual System Organ Class categories reached statistical significance.

**Table 1:** Summary of adverse events

	Risedronate	
	5-mg daily	150-mg once a month
	(*N* = 642)	(*N* = 650)
	*n* (%)	*n* (%)
AEs	554 (86.3)	578 (88.9)
Serious AEs	51 (7.9)	77 (11.8)
Deaths	4 (0.6)	0
Withdrawn due to an AE	84 (13.1)	80 (12.3)
Most common AE associated with withdrawal		
Gastrointestinal disorder	49 (7.6)	47 (7.2)
Most common AEs		
Influenza	57 (8.9)	94 (14.5)
Nasopharyngitis	62 (9.7)	70 (10.8)
Diarrhea	43 (6.7)	69 (10.6)
Arthralgia	68 (10.6)	65 (10.0)
Back pain	80 (12.5)	65 (10.0)
Bronchitis	68 (10.6)	57 (8.8)
AEs of special interest		
Clinical vertebral fracture	6 (0.9)	4 (0.6)
Nonvertebral fracture	25 (3.9)	28 (4.3)
Upper gastrointestinal tract AEs	148 (23.1)	169 (26.0)
Selected musculoskeletal AEs^a^	172 (26.8)	163 (25.1)
Atrial fibrillation	1 (0.2)	3 (0.5)
Neoplasms^b^	23 (3.6)	25 (3.8)

^a^Includes arthralgia, back pain, bone pain, musculoskeletal pain, musculoskeletal discomfort, myalgia, and neck pain

^b^Includes benign and malignant neoplasms, polyps, and cysts

*AE* adverse event

Adverse events of special interest for bisphosphonates (clinical vertebral and nonvertebral fractures, upper gastrointestinal tract adverse events, and musculoskeletal adverse events) were reported by similar proportions of subjects in both treatment groups (Table 1). The incidence of atrial fibrillation reported as either an adverse event or a serious adverse event was low and similar between groups (Table 1). There were no reported cases of osteonecrosis of the jaw. The number of subjects who developed a neoplasm did not differ by treatment group (Table 1). Results of clinical chemistry and other laboratory measurements, including measures of hepatic and renal function, were similar in both treatment groups.

## Discussion

Risedronate is a widely used osteoporosis treatment with proven vertebral and nonvertebral antifracture efficacy and a minimum wait of 30 min after dosing before eating or drinking anything other than water. A 5-mg daily regimen was developed originally, but less frequent dose regimens have now been developed. This study was a preplanned 2-year study comparing a dose of risedronate 150-mg once a month to the 5-mg daily dose. These 2-year data show that the 150-mg once-a-month dose continues to produce clinical effects that are similar to those seen with the 5-mg daily dose. Specifically, the mean percent change in lumbar spine BMD at 24 months in the monthly group was non-inferior to the mean percent change in lumbar spine BMD in the daily group. Changes in secondary efficacy parameters, including BMD at the hip, bone turnover markers at endpoint, and morphometric vertebral fractures, were also similar in both groups. Statistically significant differences between treatment groups were observed for all three bone turnover markers at month 3, but persisted at months 6 and 12 for CTX only. No statistically significant differences between groups were observed for any marker at month 24 or endpoint. That the CTX response did not differ between treatment groups at month 24 might be explained by the small number of subjects at month 24 that would limit statistical power to observe difference. It is not likely that these small differences between groups in bone turnover markers are clinically meaningful.

The risedronate 150-mg once-a-month dose was well tolerated over 2 years, with a safety profile similar to that seen with the 5-mg daily regimen. The low incidences of subjects with vertebral and nonvertebral clinical fractures were similar between groups and consistent with rates previously observed with the 5-mg daily dose [1–3].

Change in BMD is an appropriate endpoint when evaluating a new dosing schedule of a bisphosphonate for which a fracture benefit has already been established. Similar non-inferiority trials have been conducted previously to evaluate new dosing regimens of oral bisphosphonates [4, 8, 9], and this approach has been accepted by both the US Food and Drug Administration and the European Medicines Agency [10] for approval of new regimens of established agents. The magnitude of BMD change associated with the vertebral and nonvertebral antifracture efficacy of risedronate has been established in multiple large studies that had fracture as the primary endpoint [1–3]. This study has demonstrated that the 150-mg once-a-month dose reduces bone turnover and increases BMD to a degree comparable to that observed with the 5-mg daily dose in these fracture studies.

The results of this study after 2 years are consistent with the findings at month 12 [6], demonstrating the persistent similarity between risedronate 150-mg once-a-month and the 5-mg daily dosing regimens. Additionally, these results are consistent with the favorable tolerability and efficacy profiles observed in large placebo-controlled clinical trials of the risedronate 5-mg daily regimen [1–3]. The findings are also consistent with previous studies of less frequent dosing with risedronate. Such studies showed that the treatment effects of risedronate 35-mg weekly and 75-mg on two consecutive days each month were similar to the effects of daily dosing [4, 5].

Risedronate 150-mg once a month, taken for 2 years, is similar in efficacy and tolerability to the 5-mg daily dosing regimen that had been proven to reduce the incidence of vertebral and nonvertebral fractures. The addition of this dosing regimen to the therapeutic armamentarium will provide women with postmenopausal osteoporosis a full range of risedronate oral dosing options, from daily to weekly to monthly.

## Appendix

The other principal investigators in this study are: Argentina—C. Magaril, Buenos Aires; C. Mautalen, Buenos Aires; H. Salerni, Buenos Aires. Australia—G. Nicholson, Geelong, Victoria. Belgium—Y. Boutsen, Mont-Godinne; J.-P. Devogelaer, Brussels; J.-M. Kaufman, Gent. Canada—J. Brown, Quebec; D. Hanley, Calgary; W. Olszynski, Saskatoon; L.-G. Ste.-Marie, Quebec. Estonia—K. Maasalu, Tartu; K.-L. Vahula, Pärnu; I. Valter, Tallinn. Finland—J. Heikkinen, Kemi; H. Kroger, Kuopio; L. Makinen, Turku; M. Valimaki, Helsinki. France—P. Fardellone, Amiens; M. Laroche, Toulouse; G. Weryha, Vandoeuvre. Hungary—T. Hidvégi, Gyor; C. Horvath, Budapest; L. Koranyi, Balatonfüred; P. Lakatos, Budapest. Lebanon—G. E.-H. Fuleihan, Beirut. Norway—J. Halse, Oslo; E. Ofjord, Paradis; T. Sordal, Trondheim. Poland—J. Badurski, Bialystok; E. Marcinowska-Suchowierska, Warszawa; J. Pazdur, Warszawa. Spain—J. Farrerons, Barcelona; C. L. Tonkin, Madrid; M. M. Torres, Granada. USA—M. Bolognese, Bethesda, MD; R. Recker, Omaha, NE; C. Recknor, Gainesville, GA; N. Watts, Cincinnati, OH.

## Abbreviations

AE: Adverse event
BALP: Bone-specific alkaline phosphatase
BMD: Bone mineral density
CI: Confidence interval
Cr: Creatinine
CTX: C-terminal crosslinking telopeptide of type I collagen
DXA: Dual x-ray absorptiometry
ITT: Intent to treat
MedDRA: Medical Dictionary for Regulatory Activities
NTX: N-terminal crosslinking telopeptide of type I collagen
